# Levulose in the Dietetics of Childhood

**Published:** 1904-04

**Authors:** 


### ABSTRACTS.

### Levulose in the Dietetics of Childhood.

FUERST, sanitary councellor of Berlin, in a paper on the importance of levulose in the dietetics of childhood (*Zeitschrift f. diät. and physik. Therapie,* Feb. 1, 1903,) says that this pure carbohydrate of Schering has many advantages for pediatric use. It is peculiarly suited for dietetic purposes, and its value as a remedy for chemical disturbance of nutrition has not by any means been adequately appreciated. This is the more remarkable because its usefulness as a food for diabetics has been testified to by Ebstein, von Leyden, von Hebra, H. Strauss, Fr. Voit and other authorities. Ordinary cane sugar is very apt to cause dyspepsia or intestinal fermentation in nurslings; and in powder form is very apt to be adulterated. Milk sugar is not sweet enough in small amounts; in large amounts it causes diarrhea; nor can its purity be relied upon.

Levulose is a carbohydrate that has most important advantages as a sweetener of infant's food. Its molecular form and caloric value is similar to that of milk sugar; and the agreeable taste that it gives the milk renders it most suitable. Even when used for months at a time it never causes gastric or intestinal disturbance, and it is very rapidly assimilated.

Fuerst gave levulose in 17 dispensary cases, all belonging to the poorer class and with unfavorable surroundings. Three suffered from general scrofulosis (glandular), without any signs of tuberculosis; three were probably tubercular and had chronic bronchitis and bronchiolitis after influenza, measles, and pertussis and were greatly emaciated; two had anemia; and one could not recover from a severe bronchopneumonia. In all of them, as in the children not suffering from a definite disease, the dietetic-hygienic conditions were as far as possible regulated; and in all of them several weeks of levulose treatment caused visible improvement and increase in weight.

The author divides the cases in which he used the remedy into three classes and analyses its action in each of them. They are: (1) infants fed chiefly on cow's milk and suffering from malnutrition; (2) children over 2 years old suffering from insufficient nutrition, and (3) sick or convalescent children. Fuerst is not certain whether alimentary tuberculosis due to infection through the milk does occur; but there is always the possibility of bacterial invasion from the intestines. The extremely ready assimilation of levulose, however, gives a means of combatting infections of the mesenteric glands of this nature. In general in children predisposed to tuberculosis, levulose in combination with an appropriate diet will do a great deal; and it will not be less efficient because it is a simple, nonartificial and natural dietetic agent.

